# Plasmonic Properties of Individual Gallium Nanoparticles

**DOI:** 10.1021/acs.jpclett.3c00094

**Published:** 2023-02-16

**Authors:** Michal Horák, Vojtěch Čalkovský, Jindřich Mach, Vlastimil Křápek, Tomáš Šikola

**Affiliations:** † Central European Institute of Technology, Brno University of Technology, Purkyňova 123, 612 00 Brno, Czech Republic; ‡ Institute of Physical Engineering, Brno University of Technology, Technická 2, 616 69 Brno, Czech Republic

## Abstract

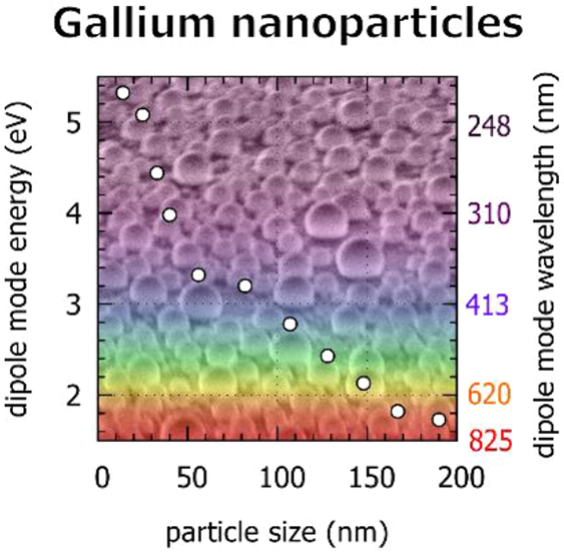

Gallium is a plasmonic material offering ultraviolet to near-infrared tunability, facile and scalable preparation, and good stability of nanoparticles. In this work, we experimentally demonstrate the link between the shape and size of individual gallium nanoparticles and their optical properties. To this end, we utilize scanning transmission electron microscopy combined with electron energy loss spectroscopy. Lens-shaped gallium nanoparticles with a diameter between 10 and 200 nm were grown directly on a silicon nitride membrane using an effusion cell developed in house that was operated under ultra-high-vacuum conditions. We have experimentally proven that they support localized surface plasmon resonances and their dipole mode can be tuned through their size from the ultraviolet to near-infrared spectral region. The measurements are supported by numerical simulations using realistic particle shapes and sizes. Our results pave the way for future applications of gallium nanoparticles such as hyperspectral absorption of sunlight in energy harvesting or plasmon-enhanced luminescence of ultraviolet emitters.

In metallic nanostructures, collective oscillations of free electrons are strongly coupled to the electromagnetic field forming the excitations called localized surface plasmons (LSP). A characteristic feature of LSP is a strong enhancement of an electromagnetic field within the surrounding dielectric together with its confinement on the subwavelength scale, which can be utilized to control various optical processes even below the free space diffraction limit.^1^ This feature is utilized in numerous applications.^2^ The most common plasmonic metals are gold and silver, but their performance is restricted at lower wavelengths by interband transitions. Consequently, gold supports LSP at wavelengths of >550 nm and silver supports LSP at wavelengths of >350 nm. The ultraviolet and whole visible spectral range is covered by aluminum,^3^ magnesium,^4^ and gallium.^5−7^ The ultraviolet plasmonic activity was theoretically studied and discussed also for bismuth, chromium, copper, indium, lead, palladium, platinum, rhodium, ruthenium, tellurium, tin, titanium, and tungsten.^8−10^ Moreover, unconventional plasmonic materials are utilized in specific application fields, including the spectro-electrochemistry prospect of silver amalgam nanoparticles^11^ or tunable plasmonic devices or metasurfaces made of phase-changing materials such as vanadium dioxide^12,13^ or gallium.^5,14,15^

Gallium is a metal with a melting temperature of 29.7 °C. It has several solid-state phases that enable a variety of phase-changing systems.^5,14,16,17^ The volume plasmon energy of gallium is 13.7 eV.^18^ In the liquid phase and the solid gamma and delta phases, gallium has a nearly Drude-like optical response from the infrared to ultraviolet spectral region, while alpha and beta phases exhibit strong interband absorption in the red and green region.^5,6,14,19^ Consequently, the former phases present an ideal broad-range plasmonic material, while the latter phases are applicable for plasmonics in the blue and ultraviolet spectral region. In addition, gallium is nontoxic and rather friendly to the environment.^20,21^ Gallium nanoparticles can be prepared by various bottom-up fabrication techniques like colloidal synthesis,^22^ optically regulated self-assembly,^23^ molecular beam epitaxy,^24^ and Joule-effect thermal evaporation.^7^ Importantly, the low melting temperature of gallium allows low-temperature fabrication with low energy consumption. Prior studies reported plasmonic properties of gallium nanoparticles,^6,25^ tuning of the plasmon resonance by oxidation creating Ga–Ga_2_O_3_ core–shell structures^26^ that were further studied in detail, including their thermal stability,^27^ gallium–indium alloy nanoparticles,^28^ and silver–gallium alloy nanoparticles.^29^ There are numerous applications for such nanoparticles. They can be used, for example, as DNA biosensing platforms,^30^ for enhancing the luminescence of MoS_2_ monolayers,^31^ for surface-enhanced Raman spectroscopy applications,^32−34^ or as the anode material for Li-ion batteries.^21^

Most of the studies of plasmonic properties of gallium addressed ensembles of nanoparticles. Only two experimental studies have focused on the optical properties of individual gallium nanoparticles. These studies utilized either electron energy loss spectroscopy^25^ or cathodoluminescence.^6^ Here, we present a study combining electron energy loss spectroscopy in a scanning transmission electron microscope (STEM-EELS) and numerical simulations to address the optical response of individual gallium nanoparticles. We show the spectral tunability of the dipole LSP mode over the near-infrared to the ultraviolet spectral range and correlate it with the size of gallium nanoparticles.

We have prepared gallium nanoparticles by direct deposition of gallium atoms onto a 50 nm thick silicon nitride membrane using a gallium effusion cell developed in house under ultra-high-vacuum conditions (see Methods). First, the samples were inspected by scanning electron microscopy (SEM). The micrographs of both samples are shown in Figure 1a–d. For the EELS analysis, we selected two samples fabricated at different temperatures. Sample A grown at a higher temperature (320 °C) contains nanoparticles with diameters in the range of 10–200 nm (Figure 1a), whereas sample B grown at a lower temperature (290 °C) contains nanoparticles with diameters in the range of 10–60 nm (Figure 1c). The average coverage of the samples by nanoparticles was determined from the SEM images and equals 50% for sample A and 70% for sample B. Considering the total Ga dose, which was larger for sample A by 50% (see Methods), the average height of the particles in sample A is ∼2 times larger than for sample B. This is in line with three-dimensional visualization of the particles obtained by SEM at tilted samples (Figure 1b,d). The nanoparticles are stable under electron beam illumination during the measurement. The three-dimensional morphology of the nanoparticles (Figure 1e–g) was determined by STEM-EELS. All of the particles have a shape similar to a lens with an aspect ratio (defined as the diameter-to-height ratio) mostly between 2.5 and 2.8 (Table 1). Consequently, our lens-shaped gallium nanoparticles are all geometrically similar.

**Figure 1:**
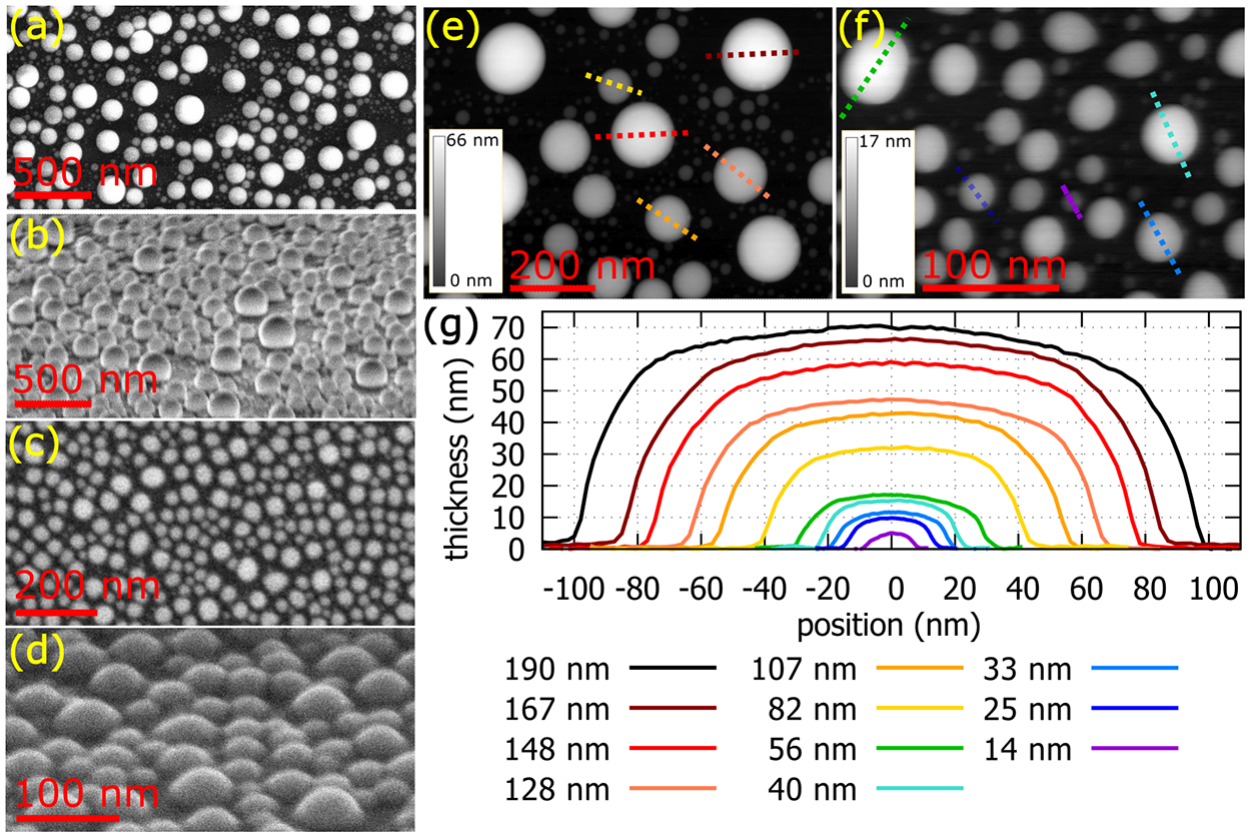
Morphology of gallium nanoparticles. (a–d) SEM images of sample A with larger nanoparticles (a and b) and sample B with smaller nanoparticles (c and d) at 0° (a and c) and 60° (b and d) tilt of the sample. (e and f) Thickness maps measured by STEM-EELS for samples A and B, respectively. (g) Thickness profiles of gallium nanoparticles in panels e and f with diameters ranging from 14 nm (violet) to 190 nm (black). Note that the largest gallium nanoparticle is not shown in panel e.

**Table 1:** Structural and Plasmonic Properties of Gallium Nanoparticles

				dipole LSP mode		
	diameter (nm)	maximum thickness (nm)	aspect ratio	energy (eV)	wavelength (nm)	*Q* factor
sample A	190	71	2.7	1.73	717	1.4
	167	66	2.5	1.82	681	1.7
	148	59	2.5	2.13	582	1.5
	128	47	2.7	2.43	510	1.9
	107	42	2.5	2.78	446	1.9
	82	32	2.6	3.20	387	3.1
sample B	56	17	3.3	3.32	370	2.1
	40	15	2.7	3.98	311	2.1
	33	12	2.8	4.44	279	1.5
	25	10	2.5	5.08	244	2.0
	14	5	2.8	5.32	233	3.1

First, we studied the crystallography and chemical composition of our gallium nanoparticles (Figure 2). According to the gallium phase diagram for nanoparticles or thin films,^14,35^ one may expect that such nanoparticles should be crystalline, consisting of the gamma phase of gallium (γ-Ga). However, the selected area diffraction pattern (Figure 2b,c) shows that our nanoparticles are amorphous or liquid. As the nanoparticles have no facets, they are supposed to be in the form of a supercooled liquid. The supercooled liquid form of colloidal Ga nanoparticles has been observed at a temperature above 250 K^22^ and the liquid phase of Ga droplets in epoxy resin is reported at a temperature above 254 or 256 K.^36,37^

**Figure 2:**
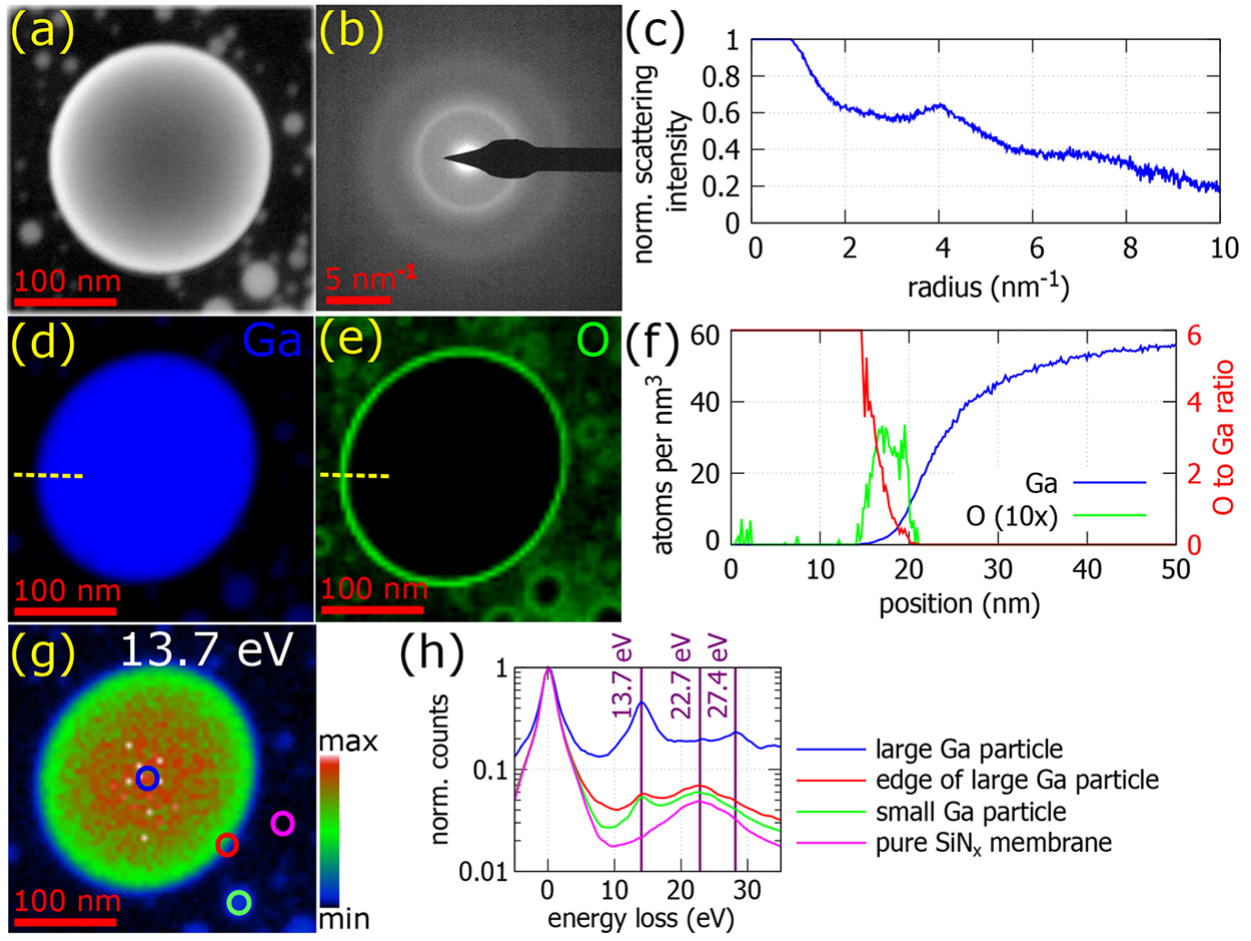
Crystallography and chemical composition of a gallium nanoparticle. (a) STEM high-angle annular dark field (HAADF) micrograph of the nanoparticle. (b and c) Selected area diffraction pattern and its rotational average, respectively, of this nanoparticle proving its amorphous character. (d and e) Chemical composition of this nanoparticle determined by STEM-EELS for the two most important elements, Ga and O, respectively. (f) Line scans of the volumetric density of Ga and O (multiplied by 10) and their ratio over the dashed yellow lines in panels d and e. (g) EELS map at an energy of (13.7 ± 0.5) eV corresponding to the volume plasmon of Ga. (h) Low-loss EEL spectra at four different positions marked in panel g.

Chemical composition was determined by both core-loss and low-loss STEM-EELS (Figure 2d–h). The nanoparticles contain pure gallium in their volume. Oxygen is another significant element present in the vicinity of the nanoparticles, in particular, next to their edges. We will discuss two possible scenarios for the role of the oxygen: (i) a thin self-terminated gallium oxide layer encapsulating the nanoparticles, also reported in ref (6), and (ii) contamination of the membrane, with the deposited gallium acting as a surface oxygen cleaner forming the oxygen-rich areas just very close to Ga nanoparticles. As oxygen is mostly concentrated next to the edges of the nanoparticles and partially on the membrane between the nanoparticles, it acts rather as a part of contamination of the membrane’s surface than as a shell part of a core–shell Ga–GaO_*x*_ structure. Were a GaO_*x*_ layer to form, it would be noticeable with a constant oxygen-to-gallium ratio, equal to 1.5 for the most usual stoichiometry Ga_2_O_3_, over several nanometers at the edge of the nanoparticle, which is not the case in Figure 2f. Moreover, a GaO_*x*_ layer would be detected by a shift of the volume plasmon peak at 13.7 eV for Ga to a different energy for GaO_*x*_, which is not the case in our low-loss STEM-EELS analysis (Figure 2g,h). Instead, we see a perfect match between the Ga elemental map (Figure 2d) determined by core-loss EELS and the energy-filtered map at 13.7 eV (Figure 2g), the energy of the Ga volume plasmon peak. Figure 2h shows the low-loss STEM-EELS spectra from four different positions on the sample: the middle of the large gallium particle, the edge of this particle, a small gallium particle, and a pure silicon nitride membrane. The first three spectra contain a peak at 13.7 eV corresponding to the Ga volume plasmon peak. All four spectra contain a peak at 22.7 eV that corresponds to the SiN_*x*_ volume plasmon peak coming from the membrane. Finally, the spectrum recorded in the middle of the large gallium particle contains a peak at 27.4 eV corresponding to the second volume plasmon peak of Ga (note that 2 × 13.7 eV = 27.4 eV). As there is no other peak visible in the low-loss EEL spectra, no other compound, except contamination, is present in the sample. Consequently, the second scenario is correct for our case, and our gallium nanoparticles are homogeneous over the whole volume and composed of high-purity gallium.

Second, we have focused on the plasmonic properties of gallium nanoparticles that are measured by STEM-EELS at room temperature. The full low-loss EEL spectrum contains the contribution of multiple scattering mechanisms and a large amount of the electrons transmitted through the sample without inelastic scattering (so-called zero-loss peak). To transform measured counts into a quantity that is proportional to the loss probability (termed loss probability in Figures 3 and 4), we divide the whole EEL spectrum by the integral intensity of the unscattered electrons. To isolate the contribution of the LSP and remove all of the other effects from EEL spectra, we define the signal spectrum as the full spectrum from which a reference spectrum is subtracted. The full spectrum is taken from the nanoparticle, and the reference spectrum is from a pure membrane. An example of the full, reference, and signal EEL spectra is shown in Figure 3. We take the raw spectrum (red) integrated over the red area in the inset and subtract the spectrum of a pure membrane (blue) to obtain the signal (green) that consists of three peaks in the low-loss region. The peak at 13.7 eV corresponds to the volume plasmon peak of gallium.^18^ The other two peaks are fitted by a Gaussian. At 7.4 eV, we observe the peak corresponding to the surface plasmon (SP) of gallium. At energies below this value, we find peaks corresponding to individual localized surface plasmon (LSP) modes. In the spectra in Figure 3, we see the peak at 3.32 eV that corresponds to a dipole mode of LSP. This is the only peak that is supposed to change its spectral position when the nanoparticle size changes. As the properties, i.e., the energy and the full width at half-maximum (fwhm), of the volume plasmon peak and surface plasmon peak remain constant, we can apply a fitting procedure, illustrated in Figure 3, which enables us to determine the energy of dipole LSP modes for the smallest nanoparticles even when they overlap with the surface plasmon peak. By fitting the spectral profile of the modes by a Gaussian, we obtained LSP resonance energy *E*, and *Q* factor, defined as the LSP resonance energy divided by its fwhm to provide a complete analysis.

**Figure 3:**
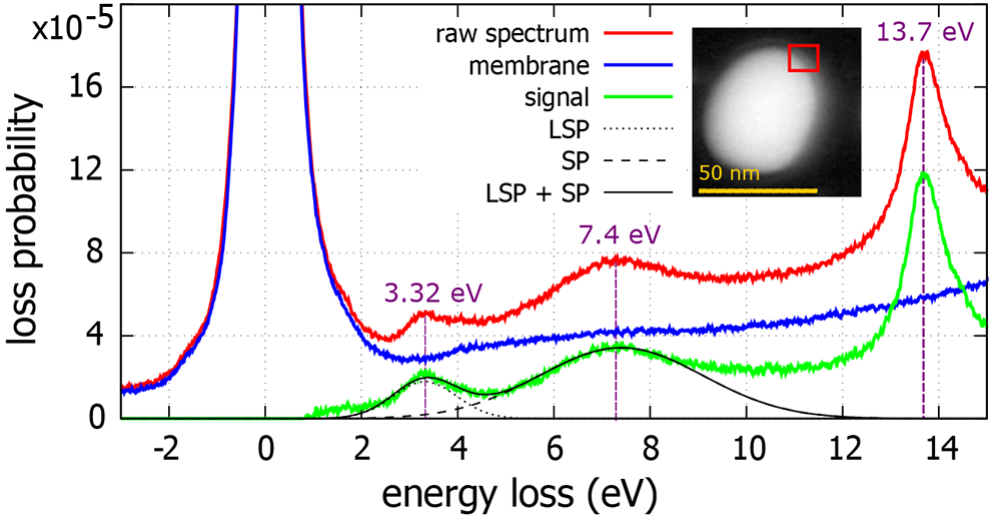
Processing of the EEL spectrum in the case of a gallium nanoparticle with a diameter of 56 nm. The low-loss EEL spectrum contains three peaks. The peak at 13.7 eV corresponds to the volume plasmon peak of gallium. At 7.4 eV, we observe the peak corresponding to the surface plasmon (SP) of gallium. At energies below this value, we find peaks corresponding to individual localized surface plasmon (LSP) modes. In this spectrum, we see the peak at 3.32 eV that corresponds to a dipole mode of LSP.

**Figure 4:**
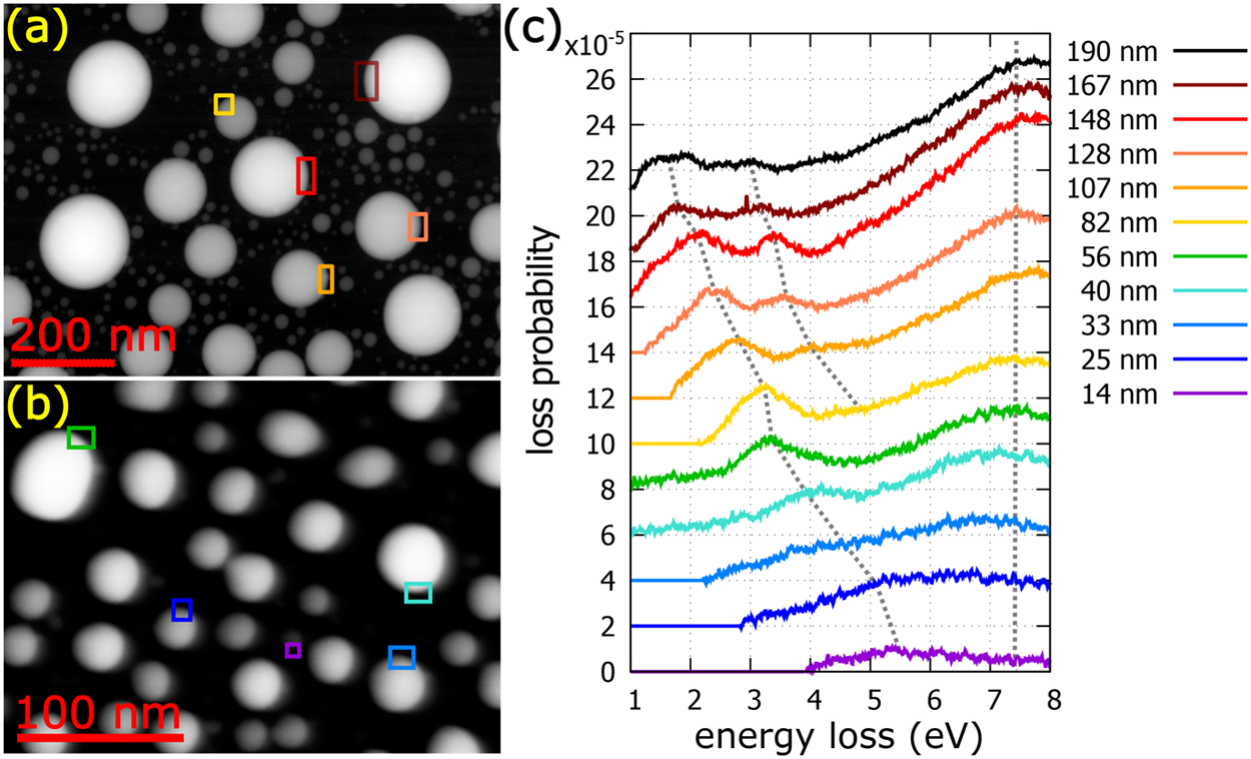
Processed EEL spectra of gallium nanoparticles. (a and b) STEM-HAADF micrograph of samples A and B, respectively. (c) Membrane-subtracted EEL spectra integrated over marked areas in panels a and b. Note that the largest gallium nanoparticle is not shown in panel a. Dashed lines in panel c are guides for the eye and follow the dipole- and quadrupole-localized surface plasmon mode whose energy changes as a function of the particle diameter and the surface plasmon in Ga at a constant energy of 7.4 eV. The morphology of these gallium nanoparticles is shown in Figure 1.

Such a complete experimental analysis of the representative nanoparticles, whose morphology is discussed in Figure 1, is shown in Figure 4. According to the expectations, the EEL spectra should contain one peak at a constant energy of 7.4 eV corresponding to the surface plasmon of gallium and one or, for larger particles, more peaks that should red-shift with an increase in nanoparticle size corresponding to the dipole and for larger particles to higher-order modes. This is exactly what Figure 4c shows. The dashed lines are guides for the eye. The first one follows the dipole LSP mode whose energy changes from 1.73 eV for the largest (190 nm in diameter) nanoparticle to 5.32 eV for the smallest (14 nm in diameter) nanoparticle as a function of the particle diameter. The second one follows the quadrupole LSP mode, which is visible just for larger particles. The third one follows the surface plasmon in gallium at a constant energy of 7.4 eV. Consequently, we have experimentally proved that the dipole LSP mode of the gallium nanoparticle can be tuned from the ultraviolet spectral region, represented by the nanoparticle with a diameter of 14 nm with the dipole resonance at 5.32 eV (corresponding wavelength of 233 nm), to the red end of the visible spectral region, represented by the nanoparticle with a diameter of 190 nm with the dipole resonance at 1.73 eV (corresponding wavelength of 717 nm). Larger gallium nanoparticles would probably support LSP resonances in the infrared spectral region. We note that our results are in agreement with a similar system studied with different methods (finite-difference time-domain numerical simulations with a partial experimental verification by cathodoluminescence) described in the literature^6^ where the dipole mode in gallium nanoparticles on a silicon substrate was found to be in the wavelength range of 200–800 nm depending on the particle size. The energy and the *Q* factor of the dipole LSP mode for all studied nanoparticles are summarized in Table 1. The *Q* factors are for most nanoparticles ∼2. Naturally, these values are influenced by instrumental broadening, but still, we can compare them with *Q* factors found in the literature. *Q* factors obtained by the same analytical method (i.e., STEM-EELS) were evaluated for gold nanorods reaching values of ∼3 for the dipole mode at a resonant energy of ∼1.1 eV.^38^

Additionally, we have performed numerical simulations to support our experimental results using the real particle sizes as the model parameters. We have performed two sets of calculations: one for liquid gallium and one for solid gallium using the dielectric functions taken from ref (6). We note that the largest differences in the dielectric functions of different Ga phases are below 2 eV, where some of the Ga phases have interband transitions.^17^Figure 5 shows the energy of the dipole LSP mode in a set of gallium nanoparticles as a function of the nanoparticles’ diameter. The nanoparticle size was determined from STEM-HAADF micrographs (shown in Figure 4a,b). The uncertainty in particle size corresponds to the size of two pixels of the image and amounts to 4–5 nm. The error bars of the energies include a standard error of the Gaussian fit to the experimental spectrum and a systematic error. The latter, primarily related to the energy width of the zero-loss peak, is estimated to be 0.1 eV. We see generally a good agreement between the experiment and the numerical simulations. However, if we consider the largest nanoparticles, we see a nonnegligible difference between the simulations for solid and liquid gallium. As our experimental values are closer to the curve for the liquid gallium, we expect that our nanoparticles are in fact in the form of supercooled liquid. This finding is in line with the selected area diffraction pattern shown in Figure 2b and the corresponding discussion. The experimental and numerical dependencies can be reasonably well approximated by the empirical power law *E*(*d*) = *A*/*d*^*n*^, where *E* is the energy of the dipole LSP mode and *d* is the diameter of the Ga particle. We note that this approximation is not valid for nanoparticles with diameters of <20 nm. The empirical parameters *A* and *n* determined from the least-squares method are listed in Table 2, and the empirical power laws together with actual energy–diameter dependencies are shown in Figure 5b. The experimental dependence is slightly steeper than the numerical one, which is also demonstrated by a slightly larger exponent *n* (0.48 for the experimental data compared to 0.33 for the numerical data with liquid gallium). This difference suggests minor inaccuracy in the determination of the particle shape, dimensions, or dielectric function utilized in the numerical model.

**Figure 5:**
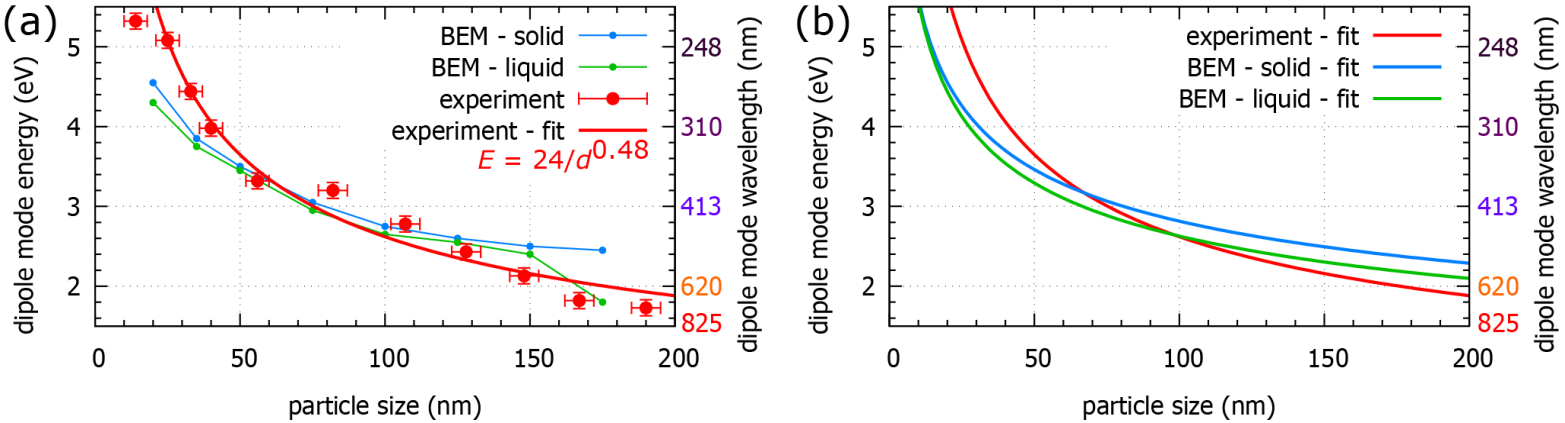
Energy of the dipole-localized surface plasmon mode in a set of gallium nanoparticles covering the spectral region from the ultraviolet across the visible to the near-infrared. (a) Comparison of experimental data obtained from the EELS measurement (red), fitted also by the empirical power law *E*(*d*) = 24/*d*^0.48^, with energy *E* expressed in electronvolts and particle diameter *d* in nanometers, and results of numerical simulations for solid (blue) and liquid (green) Ga particles. (b) Empirical power law *E*(*d*) = *A*/*d*^*n*^ fit using the least-squares method to the actual data obtained from the experiment (red) and calculated for solid (blue) and liquid (green) Ga particles.

**Table 2:** Parameters of the Empirical Power Law *E*(*d*) = *A*/*d*^*n*^^a^

data set	experiment	BEM – liquid	BEM – solid
*A*	24 ± 3	11.7 ± 1.4	11.1 ± 0.3
*n*	0.48 ± 0.03	0.33 ± 0.03	0.299 ± 0.006

a For the sake of convenience, energy *E* is expressed in electronvolts and diameter *d* in nanometers. The parameters *A* and *n* are then dimensionless.

To conclude, we have explored the plasmonic properties of gallium nanoparticles at room temperature using STEM-EELS on a single-particle level. Gallium nanoparticles with a size in the range of 10–200 nm were grown directly on the silicon nitride membrane by deposition of gallium atoms using a gallium effusion cell developed in house under ultra-high-vacuum (UHV) conditions. We have shown that their dipole mode can be tuned via their size from the ultraviolet to visible spectral region. Our results are supported by numerical simulations using the real particle sizes as the model parameters. With regard to potential applications, previous works reported that gallium nanoparticles can be used to enhance luminescence or as a biosensing platform. Our results pave the way for future applications such as hyperspectral absorption of sunlight in energy harvesting or plasmon-enhanced luminescence of ultraviolet emitters. Moreover, further understanding of their solid-to-liquid phase change will pave the way for liquid plasmonics that would not suffer from quenching on grain boundaries and impurities and consequently might afford plasmonic nanoparticles with higher *Q* factors.

## Methods

*Gallium Nanoparticle Growth*. Samples with gallium nanoparticles were prepared by direct deposition of gallium atoms onto a 50 nm thick silicon nitride membrane using a gallium effusion cell developed in house under UHV conditions. The size of the resulting nanoparticles is influenced by the temperature of the substrate, gallium flux, and time of the deposition. The samples were mounted on a bulk pyrolytic boron nitride (PBN) heater that allowed us to carefully introduce a temperature that was as constant as possible onto the whole membrane’s surface to control the size distribution of gallium droplets on the surface and their surface diffusion mobility. A higher temperate of the substrate during the deposition leads to the higher mobility of gallium at the surface. Consequently, gallium diffuses over larger distances and clusters together into larger nanoparticles. Additionally, the growth of Ga droplets on a silicon substrate is discussed in detail in ref (39). Sample A (larger nanoparticles) was deposited at 320 °C for 3 h. Sample B (smaller nanoparticles) was deposited at 290 °C for 2 h. The flux density of the gallium atoms was in both cases equal to 7.2 × 10^12^ atoms s^–1^ cm^–2^. Thus, the deposited Ga dose is larger by 50% for sample A than for sample B due to the longer deposition time. Due to the fragility of the membranes, samples were left after deposition under UHV conditions for 2 h to cool and release the tension. Both samples were prepared at a pressure of 3.8 × 10^–8^ Pa.

*Scanning Electron Microscopy*. Imaging was performed with a SEM FEI Verios instrument at a primary beam energy of 5 keV and a beam current of 50 pA using secondary electrons.

*Transmission Electron Microscopy*. Imaging, diffraction, and electron energy loss spectroscopy (EELS) measurements were performed with a TEM FEI Titan instrument equipped with a GIF Quantum spectrometer operated at a primary beam energy of 300 kV. The chemical composition of the sample was determined by a STEM-EELS measurement in dual-EELS mode to acquire the low-loss and core-loss spectra simultaneously. The spatial resolution of the EELS spectrum images is determined by the pixel size, which was set to 5 nm for the EELS maps and 0.2 nm for the EELS line scan. Such settings led to the acquisition of one spectrum image with a stable electron beam in a reasonable time. LSP resonances were measured in a monochromatic scanning regime. The beam current was set to 0.1 nA, and the full width at half-maximum of the zero-loss peak (ZLP) was ∼0.15 eV. We set the convergence semiangle to 10 mrad, the collection semiangle to 14.4 mrad, and the dispersion of the spectrometer to 0.01 eV/pixel. These parameters were selected to acquire sufficient EELS signal over the areas with a large change in thickness.^40^ The acquisition time was adjusted to use the maximal intensity range of the CCD camera in the spectrometer and avoid its overexposure. The spatial resolution of the EELS spectrum images is determined by the pixel size, which was set to 2 nm. To reduce the noise in the LSP signal, the EEL spectra were integrated over the rectangular areas at the nanoparticle edges (marked in Figure 4) where the LSP resonance is significant. They were further divided by the integral intensity of the ZLP to transform measured counts to a quantity proportional to the loss probability (termed loss probability in the EEL spectra in Figures 3 and 4), membrane subtracted, and fitted by Gaussians (see Figure 3). The thickness of the Ga nanoparticles was evaluated from the low-loss EELS in terms of relative thickness, which is proportional to the absolute thickness with the inelastic mean free path (IMFP) as the constant of proportionality. The IMFP in gallium for the actual parameters of the electron beam (electron energy of 300 keV and collection semiangle of 14.4 mrad) was calculated using the software package EELSTools by Mitchell^41^ applying the algorithm of Iakoubovskii et al.^42^ and equals 156 nm. By multiplying the relative thickness by this value, we obtain the approximate absolute thickness of Ga nanoparticles.

*Simulations*. Numerical simulations of EEL spectra were performed using the MNPBEM toolbox^43^ based on the boundary element method (BEM). The structures were modeled as gallium nanodiscs with a rounded upper edge and an aspect ratio of 2.5. The dielectric function of solid and liquid gallium was taken from ref (6), and the dielectric function of the silicon nitride membrane was set to 4, which is a common approximation in the considered spectral region.^44^ The 300 keV electron beam was situated outside the nanodisc 5 nm far from its edge. In addition to the nanodisc model shape, we also performed simulations for the realistic shape of the structures. To this end, we utilized the thickness distributions shown in Figure 1, which were subsequently rotated to form particles with a cylindrical symmetry. The energies of the LSP resonances corresponded rather well to those obtained for the model nanodisc shape, verifying thus its plausibility. At the same time, the realistic shape simulations suffered from numerical instabilities, which led us to prefer the model shape.

## Supporting Information Available

Transparent Peer Review report available (PDF)
