# Supplementary material for: Plasmonic Properties of Individual Gallium Nanoparticles
Source: J Phys Chem Lett. 2023 Feb 16;14(8):2012–9. doi: 10.1021/acs.jpclett.3c00094 (PMC10017019; doi:10.1021/acs.jpclett.3c00094)
Supplement: Supplementary file 1 — jz3c00094_si_001.pdf [file jz3c00094_si_001.pdf]

jz-2023-000943.R1

Name: Peer Review Information for "Plasmonic Properties of Individual Gallium Nanoparticles"

First Round of Reviewer Comments

Reviewer: 1

Comments to the Author

The paper is well-written, it includes high quality results and the methods are well described. However, there are some points that should be clarified.

- Firstly, if I am right, Figure 1 a) to d) is not commented in any place in the manuscript. Please, describe these SEM images.

-Secondly, I do not understand the necessity to evaporate at two different temperatures. In the Methods section it is suggested that the higher temperature, the higher the nanoparticle size. However, I guess that since the gallium atoms flux density is constant, the volume should be the same in both samples. Thus, the separation between nanoparticles should be different. It can be partially seen in figure 1 a) and c) but authors should quantify it. I recommend to try to calculate the coverage of a given image size, the number of nanoparticles and estimate a mean separation.

-Once it is done, how the plasmonic coupling between nanoparticles can affect a STEM-EELS measurement? I guess that the resolution it is so high that it can be avoided. In that case, authors should use this argument to motivate the work. Far-field optical measurements are clearly affected by plasmonic coupling while single-nanoparticle optical measurements don´t. That is a clear advantage.

-In page 7, I understood that the liquid state is attributed to the fabrication temperature. There are several reports that grow the nanoparticles at RT and they are also liquid inside. This is due to the supercooled-state of Ga in the form of nanostructures. See: doi.org/10.1021/ja506712d | J. Am. Chem. Soc. 2014, 136, 12422−12430

-In page 3 the authors state that gallium have almost no interband transitions but it is only true at liquid state. Solid phases have them. Check your reference 6th from Knight.

-In page 4 it is stated that gallium oxide has a Ga_2O_3 stoichiometry. In reference 27th it was not specified. However, it was confirmed later by XPS in the next paper: 2018 Nanotechnology 29 355707, DOI 10.1088/1361-6528/aacb9

-Could you explain what is the "y" axis "loss probability" in figure 3? or how was it calculated?

-References 7th and 25th are the same.

Reviewer: 2

Comments to the Author

The manuscript is very well written and an excellent piece of work. There are only two remarks, which have to be addressed in the revised version: (1) For the experiment, errorbars are needed (for the particel size as well as for the dipole mode energy). (2) Additionally, a fit through the experimental points in Fig. 5 should be included. This will lead to a empirically determined law for the size-wavelngth relationship. The same has to b be done with the simulated date. Differences in the fit-functions should then also be discussed.

Author's Response to Peer Review Comments:

Dear Prof. Editor,

we thank you for handling our manuscript and both Reviewers for their very useful comments, which we thoroughly utilized to improve the manuscript, and we are glad to submit a revised version of our manuscript. Point-by-point response is in the attached file.

Further, we have uploaded the manuscript pdf with all changes made during th revision in red color as the Supplementary material for Review only.

We made the requested editorial changes, too. We capitalized the first letter of each principal word in the Title and checked the formatting of References.

Thank you for your consideration.

Sincerely,

Michal Horák

on behalf of all authors

Point-by-point response to Reviewer comments

We thank both Reviewers for their very useful comments, which we thoroughly utilized to improve the manuscript, and we are glad to submit a revised version of our manuscript. Point-by-point response to Reviewer comments follows. Reviewers' reports are in black colour, our comments in blue colour, and corresponding changes in the manuscript in green colour.

Reviewer 1

The paper is well-written, it includes high quality results and the methods are well described. However, there are some points that should be clarified.

- Firstly, if I am right, Figure 1 a) to d) is not commented in any place in the manuscript. Please, describe these SEM images.

We have added a short description referring to SEM Images including the average coverage of the surface by the nanopatricles.

Modified text: *„First, the samples were inspected by scanning electron microscopy (SEM). The micrographs of both samples are shown in Figure 1a-d. For the EELS analysis, we selected two samples fabricated at different temperatures. Sample A grown at a higher temperature (320°C) contains nanoparticles with diameters in the range from 10 nm to 200 nm (Figure 1a), whereas sample B grown at a lower temperature (290°C) contains nanoparticles with diameters in the range from 10 nm to 60 nm (Figure 1c). The average coverage of the samples by nanoparticles was determined from the SEM images and equals to 50 % for sample A and 70 % for sample B. Considering the total Ga dose, which was larger for sample A by 50 % (see Methods), the average height of the particles in sample A is about twice larger than for sample B. This is in line with three-dimensional visualization of the particles obtained by SEM at tilted samples (Figure 1b,d).“*

-Secondly, I do not understand the necessity to evaporate at two different temperatures. In the Methods section it is suggested that the higher temperature, the higher the nanoparticle size. However, I guess that since the gallium atoms flux density is constant, the volume should be the same in both samples. Thus, the separation between nanoparticles should be different. It can be partially seen in figure 1 a) and c) but authors should quantify it. I recommend to try to calculate the coverage of a given image size, the number of nanoparticles and estimate a mean separation.

We have determined the average coverage of the surface by the nanoparticles from the SEM images. The mean separation was not evaluated as it is not a crucial parameter for this study dealing with individual and isolated nanoparticles.

The volume of deposited Ga is not the same as the deposition time differs as it is mentioned in Methods (2 h for 290°C vs. 3 h for 320°C). A higher temperate of the substrate during the deposition leads to the higher mobility of Ga at the substrate's surface. Consequently, Ga moves over larger distances and clusters together into larger nanoparticles.

The growth of Ga droplets on a silicon substrate is discussed in detail in Reference [10.1088/0957-4484/19/47/475606], which we added to the Methods section.

Additional explanation added to the Methods section: *„A higher temperate of the substrate during the deposition leads to the higher mobility of gallium at the surface. Consequently, gallium diffuses over larger distances and clusters together into larger nanoparticles. Additionally, the growth of Ga droplets on a silicon substrate is discussed in detail* in detail by Kolíbal et al. *[10.1088/0957-4484/19/47/475606]"…"Thus, the deposited Ga dose is larger by 50 % for sample A as compared to sample B due to longer deposition time."*

-Once it is done, how the plasmonic coupling between nanoparticles can affect a STEM-EELS measurement? I guess that the resolution it is so high that it can be avoided. In that case, authors should use this argument to motivate the work. Far-field optical measurements are clearly affected by plasmonic coupling while single-nanoparticle optical measurements don´t. That is a clear advantage.

The excitation of LSP by a focused electron beam is indeed highly localized. Consequently, we address individual nanoparticles. Naturally, we cannot exclude plasmonic interaction between closely spaced particles. Even for local excitation, the charge oscillations are excited in the whole particle, and the related near field spreads tens to hundreds of nanometers from the particle and interacts with other particles within this range. However, STEM allows to select particles isolated enough from their neighbors so that the coupling is negligible. We estimate that already for the distance above 30 nm the coupling affects the energy less than the magnitude of the spectral resolution of EELS.

-In page 7, I understood that the liquid state is attributed to the fabrication temperature. There are several reports that grow the nanoparticles at RT and they are also liquid inside. This is due to the supercooled-state of Ga in the form of nanostructures. See: doi.org/10.1021/ja506712d | J. Am. Chem. Soc. 2014, 136, 12422−12430

We thank the reviewer for this hint. We removed the sentence *“This observation can be attributed to a different fabrication process which is in our case done at the temperature of 290°C or 300°C whereas the gallium phase diagrams in the literature are for low-temperature depositions done at temperatures below 30°C.“* from the text. We added the suggested reference (no. 22) and slightly modified the corresponding text to: *“As the nanoparticles have no facets, they are supposed to be in the form of a supercooled liquid. The supercooled liquid form of colloidal Ga nanoparticles has been observed at a temperature above 250 K[22] and the liquid phase of Ga droplets in epoxy resin is reported at a temperature above 254 K or 256 K, respectively.[36,37]”*

-In page 3 the authors state that gallium have almost no interband transitions but it is only true at liquid state. Solid phases have them. Check your reference 6th from Knight.

There are many references with dielectric functions of different Ga phases which are rather different. We modified the text to be more accurate.

The modified text is: *„In the liquid phase and the solid gamma and delta phases, gallium has a nearly Drude-like optical response from infrared to ultraviolet spectral region, while alpha and beta phases exhibit strong interband absorption in the red and green.[5,6,14,19] Consequently, the former phases present an ideal broad-range plasmonic material, while the latter phases are applicable for plasmonics in blue and ultraviolet spectral region.“*

-In page 4 it is stated that gallium oxide has a Ga2O3 stoichiometry. In reference 27th it was not specified. However, it was confirmed later by XPS in the next paper: 2018 Nanotechnology 29 355707, DOI 10.1088/1361-6528/aacb9

Here, we kindly disagree with the Reviewer that Reference 27 [10.1088/1361-6528/aa8505] does not specify the Ga2O3 stoichiometry. We list in the following three sentences from this paper: „When NPs are exposed to air, a 0.5–3 nm thick passivating layer of gallium oxide (Ga_2O_3) is formed preserving the liquid Ga from the environment.“ „In order to confirm the increase of the Ga_2O_3 layer in more detail, *ex situ* XPS measurements were performed after the annealing.“ „The increase of the Ga_2O_3 thickness has been demonstrated by XPS measurements.“

Nevertheless, we thank for the new Reference [10.1088/1361-6528/aacb91], which we included in the introductory part.

Modified text in the introductory part: *"…tuning of plasmon resonance by oxidation creating Ga-Ga_2O_3 core-shell structures [10.1088/1361-6528/aa8505] which were further studied in detail including their thermal stability [10.1088/1361-6528/aacb91]…"*

-Could you explain what is the "y" axis "loss probability" in figure 3? or how was it calculated?

The loss probability is a standard quantity that is used in EELS. It is a probability of an inelastic scattering event with a certain energy loss (in the experiment corresponding to the energy width of a pixel on a CCD camera in the spectrometer). The EEL spectrum is divided by the integral intensity of the ZLP to transform measured counts on a CCD camera to a quantity proportional to the loss probability (referred to as loss probability in the EEL spectra in Figures 3 and 4). We note that it was already explained in the Methods section, but we understand that it might not be enough.

We added the explanation also into the main text before Figure 3. Text added: „*To transform measured counts into a quantity proportional to the loss probability (referred to as loss probability in Figures 3 and 4) we divide the whole EEL spectrum by the integral intensity of the unscattered electrons.*"

We added *"(referred to as loss probability in the EEL spectra in Figures 3 and 4)"* into the Methods.

-References 7th and 25th are the same.

We thank Reviewer for this information. We removed the doubled reference and renumbered the references accordingly.

Reviewer 2

The manuscript is very well written and an excellent piece of work. There are only two remarks, which have to be addressed in the revised version: (1) For the experiment, errorbars are needed (for the particel size as well as for the dipole mode energy). (2) Additionally, a fit through the experimental points in Fig. 5 should be included. This will lead to a empirically determined law for the size-wavelngth relationship. The same has to b be done with the simulated date. Differences in the fit-functions should then also be discussed.

We have added the error bars to the experimental data in Figure 5 and we have fitted the data points by an empirical power law $E(d) = A/d^n$, where E is the energy of the dipole LSP mode and d is the diameter of the Ga particle.

New text added: „*The nanoparticle size was determined from STEM-HAADF micrographs (shown in Figure 4a,b). The uncertainty in particle size corresponds to the size of two pixels of the image and amounts to 4–5 nm. The error bars of the energies include a standard error of the Gaussian fit to the experimental spectrum and a systematic error. The latter, primarily related to the energy width of the zero-loss peak, is estimated to be 0.1 eV.*"

New text added: „*Both the experimental and numerical dependences can be reasonably well approximated by an empirical power law $E(d) = A/d^n$, where E is the energy of the dipole LSP mode and d is the diameter of the Ga particle. We note that this approximation is not valid for nanoparticles with a diameter below 20 nm. The empirical parameters A and n determined from the least square method are shown in Table 2 and the empirical power laws together with actual energy-diameter dependencies are shown in Fig 5(b). The experimental dependence is slightly steeper than the numerical one, which is also demonstrated by a slightly larger exponent n (0.48 for the experimental data compared to 0.33 for the numerical data with liquid gallium). This difference suggests minor inaccuracy in the determination of the particle shape, dimensions, or dielectric function utilized in the numerical model.*"

Figure 5 was updated. Table 2 which summarizes the fitted parameters was added.
